# Cultivating the uncultured: Harnessing the “sandwich agar plate” approach to isolate heme‐dependent bacteria from marine sediment

**DOI:** 10.1002/mlf2.12093

**Published:** 2024-01-18

**Authors:** Jing Zhang, Qi‐Yun Liang, Da‐Shuai Mu, Fengbai Lian, Ya Gong, Mengqi Ye, Guan‐Jun Chen, Yuqi Ye, Zong‐Jun Du

**Affiliations:** ^1^ State Key Laboratory of Microbial Technology, Institute of Microbial Technology Shandong University Qingdao China; ^2^ Marine College Shandong University Weihai China; ^3^ Shandong University‐Weihai Research Institute of Industrial Technology Weihai China

**Keywords:** cultivation, growth factors, heme, sandwich agar plate, uncultured bacteria

## Abstract

In the classical microbial isolation technique, the isolation process inevitably destroys all microbial interactions and thus makes it difficult to culture the many microorganisms that rely on these interactions for survival. In this study, we designed a simple coculture technique named the “sandwich agar plate method,” which maintains microbial interactions throughout the isolation and pure culture processes. The total yield of uncultured species in sandwich agar plates based on eight helper strains was almost 10‐fold that of the control group. Many uncultured species displayed commensal lifestyles. Further study found that heme was the growth‐promoting factor of some marine commensal bacteria. Subsequent genomic analysis revealed that heme auxotrophies were common in various biotopes and prevalent in many uncultured microbial taxa. Moreover, our study supported that the survival strategies of heme auxotrophy in different habitats varied considerably. These findings highlight that cocultivation based on the “sandwich agar plate method” could be developed and used to isolate more uncultured bacteria.

## Impact statement

Digging into uncultured microbes has been one of microbiology's most active research topics. In this study, we designed a simple and efficient coculture system to isolate uncultured microorganisms, and many uncultured commensal bacteria in marine sediment were successfully isolated. Our findings suggest that heme is a key growth factor mediating special microbial interactions and provides insight into the potential niche partitioning factors of different heme auxotrophy. Our approach constructs a model for exploring commensal microbes. This study also provides a new train of thought to test and mine the potential ecological functions of rare species.

## INTRODUCTION

The cultivation and isolation of bacteria through pure culture methods have been vital in the development of microbiology. Nutrient agar and other derived cultured media have been, and will continue to be, employed for the culturing and isolating of microbes from various environments^1^, ^2^. However, one of the most significant issues preventing the isolation of most prokaryotic taxa is the disruption of symbiotic or other beneficial interactions between microorganisms during pure culture or isolation strategies^1^, ^2^, ^3^, ^4^. Indeed, in natural environments, most microbial cells work together and communicate in many ways (e.g., sharing metabolic substances, growth factors, chelating agents, and signaling molecules)^1^, ^2^. These complex relationships between bacteria are hardly reproducible in monocultures^1^. Hence, fully considering the interaction between microorganisms during the culturing process is crucial for the isolation and culture of the mutual and commensal microorganisms^5^.

Recognizing the fact that bacterial species rarely live in isolation, diverse coculture techniques have been developed, including (i) cocultivation of adjacent colonies^6^, ^7^, ^8^, (ii) cocultivation of microbial subcommunities in microfluidic droplets (usually 2–3 cells)^9^, (iii) medium optimization by adding spent‐culture supernatant of candidate helper bacteria^10^, (iv) the lawn culture by providing a uniform layer of bacterial growth on a solid medium^11^, and (v) cocultivation devices based on dialysis membranes, such as transwell culture chamber, Davis U‐tube, and microbial culture chamber^12^. Using these coculture techniques, scientists obtained many previously uncultured bacteria^6^, ^7^, ^8^, ^10^. They identified diverse growth factors, including iron‐chelating siderophores^6^, quinones^7^, GABA^8^, and small molecule metabolites produced by “helper organisms” (e.g., vitamin B_5_, uracil, cytosine, and IAA^10^).

While many novel species have been isolated by the above coculture methods^7^, ^8^, ^10^, most of these methods have mainly focused on the microbial interactions during the culture process rather than those of the isolation process. The main consequence of this has been a disruption of inter‐ and intra‐species communication at the beginning of isolation, which has led to difficulties in obtaining pure cultures of some bacteria that are strictly dependent on other bacteria^1^. In addition, some growth factors and metabolic intermediates with a short life or poor water solubility (e.g., heme, menaquinone, ubiquinone, polysulfides, and thiosulfate) could not be continuously supplied to the isolates by helper supernatant^7^, ^13^. Therefore, it is necessary to design a coculture method for maintaining interactions among the helper(s) and the isolates during the entire cultivation and isolation process.

It is well known that heme, an iron‐containing porphyrin ring, could serve as a cofactor in diverse proteins, including cytochromes, hemoglobin, catalases, and nitric oxide synthases^14^. Heme is a main electron carrier in the electron transport chain and essential for cellular respiration^14^. Therefore, it was generally believed that only a few host‐dependent bacteria and lactic acid bacteria lacked heme biosynthetic pathways^15^, ^16^, ^17^ and that almost all microorganisms in natural habitats could synthesize heme by themselves^16^, ^18^. Recently, Kim et al. isolated acI lineage from the basal culture by supplying catalase as a critical growth supplement and proved that acI bacteria require heme, an essential cofactor of catalase, for their growth, rather than the H_2_O_2_ scavenging function of catalase; meanwhile, their findings indicate that heme auxotrophy is a more common phenomenon than previously thought, and may lead to use of heme as a growth factor to increase the cultured microbial diversity^19^. However, there is little study about isolating the heme auxotrophies through microbial interaction.

**Figure 1:**
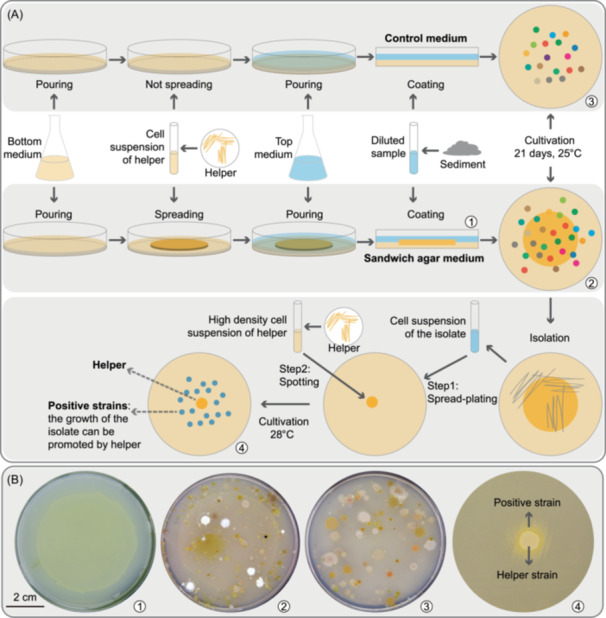
Overview of microbial isolation through sandwich agar plate method. (A) Preparation of control medium and sandwich agar media for isolation spread plates of bacteria from sediment samples. Purification and subcultivation of the isolates through their respective isolation medium and growth‐promotion assay of the isolate from sandwich agar media were performed. The isolate was spread over the entire marine agar medium, and 2 μl culture of the helper was then spotted on the same plate. If the isolate only grew or displayed improved growth around the spotted helper, it was marked as a positive strain. (B) The 1, 2, 3, and 4 marked in circle represent the blank sandwich agar plate, spread sandwich agar plate, spread control plate, and growth induction of a positive strain by a helper strain, respectively.

In this study, we developed an innovative coculture technique named the “sandwich agar plate method” (Figure 1). This technology allows for continuous interaction between living helpers and isolates, whether in the culture or isolation process. We selected rare species as helpers to cocultivate commensal bacteria. Our study provides an effective cocultivation method with excellent potential for isolating and cultivating uncultured bacteria. Meanwhile, we first isolated the heme auxotrophies through microbial interaction and proved that heme could mediate the microbial interaction during the coculture. Furthermore, we studied the difference in the distribution, life strategies, and the adaption to heme concentration of heme auxotrophies in different habitats.

## RESULTS

### Establishment of the sandwich agar plate method and the determination of helper organisms

The sandwich medium (Figure 1A) consisted of three layers: (i) the bottom medium, the main function of which is to maintain the growth of helper organisms; (ii) the cell layer of the living helper strain, which was expected to provide a continuous supply of diverse growth factors and signal molecules for the coculturing of isolates; and (iii) the top medium, that mainly acts as the growth medium for the cocultured isolates, and as a physical barrier between the isolate and helper cells. In the experiment, adjusting the bacterial coverage area of the helper organisms to a 6–7 cm circular area (plate diameter was 9 cm) and adjusting the agar concentration of the top medium to 4% could effectively prevent the helper organisms from growing through the top medium. Finally, a successful sandwich plate was created (Figure 1B). Our method allows for the interaction between isolates and helper organisms throughout the incubation and isolation process. A control medium without helper organisms was set up to test the effectiveness of these sandwich agar plates (Figure 1A).

In this study, rare species were selected as helpers (rare species: relative abundances below 0.01%^20^). It is reported that rare species can maintain and regulate community structure, function, and stability by facilitating other community members^21^; meanwhile, Rivett and Bell reported that there were more negative statistical interactions among abundant phylotypes, whereas more positive interactions between rare phylotypes^22^. Therefore, we hypothesized that the majority of rare species may play an essential role in isolating and cultivating uncultured microbes through microbial interaction. In the previous experiment, we isolated some microbes from the offshore zone sediments (at Jingzi Port of Weihai, China) multiple times. According to the high‐throughput sequencing data, the abundances of many isolated microbes were very low (below 0.01%) (Tables S1 and S2). Then, we randomly selected 8 low‐abundant species (*Colwellia aestuarii*, *Roseibacterium beibuensis*, *Formosa spongicola*, *Shewanella decolorationis*, *Marinobacter algicola*, *Salipiger mucosus*, *Cribrihabitans marinus* and *Algibacter pectinivorans*) from the cultivated rare species mainly based on the better growth status in the sandwich plates. For increasing the universality of this method, we collected those type species *C. aestuarii* KCTC 12480^T^ (S08), *R. beibuensis* MCCC 1F00103^T^ (S11), *F. spongicola* KCTC 22662^T^  (S20), *S. decolorationis* JCM 21555^T^ (S26), *M. algicola* DSM 16394^T^ (S47), *S. mucosus* LMG 22090^T^ (S60), *C. marinus* JCM 19401^T^ (S63) and *A. pectinivorans* JCM 17107^T^ (S64) as the helper strains. According to the helpers' designation numbers, the sandwich media supplemented with different helper strains were named S08, S11, S20, S26, S47, S60, S63, and S64 medium, respectively.

### Diversity of bacterial isolates from “sandwich agar media” versus control medium

**Figure 2:**
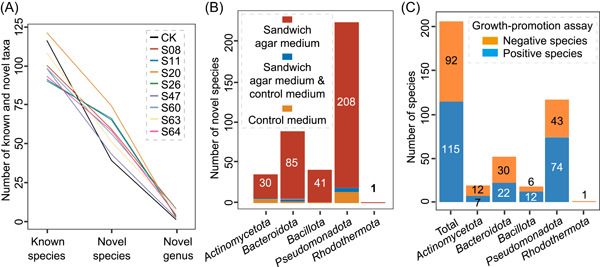
Isolation efficacy of previously uncultured bacteria on sandwich agar plates. (A) The novel species and genera isolated from sandwich agar plates (eight kinds of helper strains) and control medium. Known species, novel species, and novel genera were defined by 16S rRNA gene sequence similarity thresholds of 98.66%–100%^23^, 95.31%–98.65%^23^, and 90.00%–95.30%^25^, respectively. The lines of eight kinds of helper strains and the control were randomly colored. (B) Distribution of all novel species based on phylum. (C) Distribution of 207 species displaying positive and negative results, based on phylum, in growth‐promotion assay. A positive result indicates species' growth could be promoted by helper organisms, while species that could not be promoted were marked as negative. Helper strains, S08: *Colwellia aestuarii*; S11: *Roseibacterium beibuensis*; S20: *Formosa spongicola*; S26: *Shewanella decolorationis*; S47: *Marinobacter algicola*; S60: *Salipiger mucosus*; S63: *Cribrihabitans marinus*; S64: *Algibacter pectinivorans*.

In the experiments, offshore sediment samples were collected from the Jingzi Port of Weihai. A total of 1743 isolates, representing approximately 190 strains from each medium, were obtained (Table S3). According to a 16S rDNA sequence similarity of 98.65%^23^ or genomic average nucleotide identity value of 95%–96%,^24^ these isolates could be divided into 828 species (Tables S3 and S4). The number of novel species on the sandwich agar medium based on eight kinds of helper strains was higher than that on the control medium (Figure 2A), and there were 365 novel species (Figure 2B) and 34 novel genus isolated from the sandwich agar medium based on eight kinds of helper strains. Almost all candidate species could be assigned to four common phyla, with only a single species isolated from the S11 medium assigned to the phylum *Rhodothermota* (Table S3). Interestingly, almost all novel strains (41/42) within the phylum *Bacillota* were isolated from the sandwich agar plates (Figure 2B). These results indicated that cocultivation with rare species as helpers could substantially increase the isolation of uncultured bacteria even when utilizing the most common marine agar medium (MA). In addition, we used high‐throughput 16S rRNA gene sequencing to analyze bacterial community compositions of the sandwich agar plates with different helper organisms and without helpers (Table S5). The results showed that helper organisms could significantly alter the overall bacterial community composition on the agar plates, and the different helpers had different effects (Figure S1). Moreover, members of the low‐abundance phyla *Thermodesulfobacteriota*, *Bdellovibrionota*, *Deinococcota*, *Cyanobacteria*, and *Gemmatimonadota* were only detected in the sandwich plate group (Table S5).

To verify that the helper organisms had actual growth‐promoting effects on the isolates, 207 of the novel species (randomly selected) isolated from sandwich agar plates were cocultured with helper organisms. A diagram of the growth‐promotion assay is shown in Figure 1A. The results showed that helper organisms could significantly promote growth for more than 50% of the test species (Figure 2C and Table S6). The eight helper bacteria had different growth‐promoting effects on taxa from the four common phyla (Figure S2). As expected, most novel species of *Bacillota* were dependent on helpers for growth (Figure 2C). The most effective growth‐promoting helper was *C. aestuarii* S08, followed by *F. spongicola* S20 and *S. decolorationis* S26 (Figure S2A and Table S6). To confirm whether it works in the liquid condition, we employed the transwell to coculture the helper strains and helped strains (Figure S2B), and we found that the helper *C. aestuarii* S08 and *S. decolorationis* S26 promoted the helped strains F26177, F26174, S0825, and S0848 in the liquid media (Figure S2C). Subsequently, we determined extracellular low‐molecular‐weight organic substances (LMWOS) produced by these three helper organisms (Figure S3). The results showed that the helpers could secrete many known growth‐promoting factors (e.g., riboflavin^26^, protoporphyrin^27^, and siderophores^6^), signal molecules (e.g., indole^10^), critical synthetic intermediates, and diverse organic carbon and organic nitrogen compounds (Figure S3), which may be responsible for the culturability and growth promotion of some uncultured bacteria on the sandwich agar plates. Furthermore, we noted that multiple helpers could promote the growth of the same species, and each helper organism could promote the growth of many different species (Figure S2). It is conceivable that differences in the supplied metabolites could cause the differences in which taxa were promoted by different helper organisms (Figure S3).

### Heme serves as a growth factor for marine bacteria

**Figure 3:**
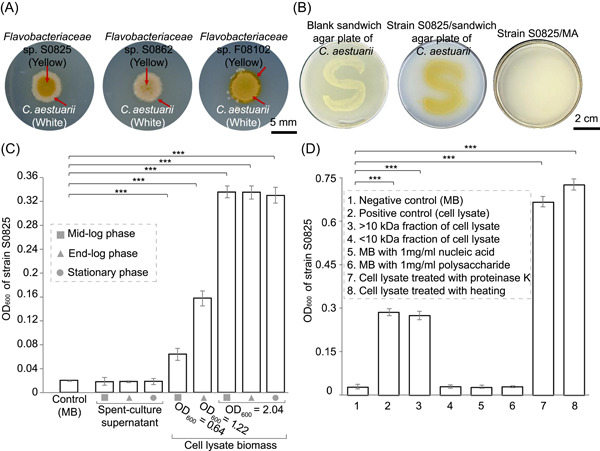
Growth of *Flavobacteriaceae* sp. S0825, S0862, and F08102 induced by *Carcinus aestuarii*. (A) The growth‐promotion assay of strains S0825, S0862, and F08102. Strain S0825, S0862, and F08102 were spread over the entire marine agar medium plate, respectively, and 2 μl culture of helper *C. aestuarii* (S08) was then spotted on the plate. Strains S0825, S0862, and F08102 only grew within the growing area of *C. aestuarii*. (B) Growth of strain S0825 on “S”‐shaped sandwich agar plate of *C. aestuarii*. Strain S0825 was spread over the entire plate and only grew above the “S”‐shaped area. (C) Growth‐promoting effects of spent‐culture supernatant and cell lysate solution of *C. aestuarii* on strain S0825. The cell lysate biomass represents the biomass of bacterial suspension before cell lysis. (D) Growth‐promoting effects of polysaccharide, nucleic acid, and cell lysate with disrupted protein structure on strain S0825. Polysaccharides and nucleic acids were extracted from the cell lysate of *C. aestuarii*. All experiments were conducted in triplicate. Error bars represent standard errors. The results of analysis of variance with Turkey's HSD tests among the groups are shown, ***p ≤ 0.001.

*C. aestuarii* S08 had the most significant growth‐promoting ability for dependent isolates (Figure S2). Therefore, we selected three isolates (*Flavobacteriaceae* sp. S0825, S0862, and F08102) strictly dependent on helper *C. aestuarii* S08 for growth‐promoting factor studies. In the growth‐promotion assay (Figure 1A), the three strains showed almost no growth in areas of the plate without helper coverage (Figure 3A). Similarly, on the sandwich plates, strain S0825 only grew above the growth area of the helper (Figure 3B). In contrast, many other dependent species, such as *Bacillaceae* species, were able to grow in the larger area around the helpers (Figure S4). These findings suggested that close intercellular distance was required for the interaction between the three *Flavobacteriaceae* species and *C. aestuarii*, and the three strains may share the same growth‐promoting mechanism. Using strain S0825 as the object, we conducted experiments to determine the potential growth factor. Spent‐culture supernatant of *C. aestuarii* did not promote the growth of strain S0825, while the cell lysate did (Figure 3C). Next, we used centrifugal filter devices with a 10 kDa cutoff to ultra‐filter the cell lysate. Only the fraction >10 kDa had growth‐promoting activity, which indicated that the growth factor was a biological macromolecule (Figure 3D). We then concentrated the >10 kDa fraction of the supernatant. The 100‐fold concentrated supernatant could promote the growth of strain S0825, which confirmed the concentration of growth factor in the supernatant may be low and unable to meet the minimum requirement of strain S0825 (Figure S5). Growth‐promoting activities of known biomacromolecules (typically polysaccharides, nucleic acid, and proteins) were then verified. Crude extracts of polysaccharides and nucleic acids from *C. aestuarii* cell lysate did not promote the growth of strain S0825 (Figure 3D). The cell lysate with protein structure disrupted by proteinase K and heating retained growth‐promoting activity, and the activity was increased by 2‐fold compared with the untreated cell lysate (Figure 3D). These results indicated that growth factor might be protein‐related, and the destruction of protein structure was beneficial to the release of this growth factor. Therefore, we speculated that the growth factor might be a prosthetic group or a cofactor tightly bound to proteins.

**Figure 4:**
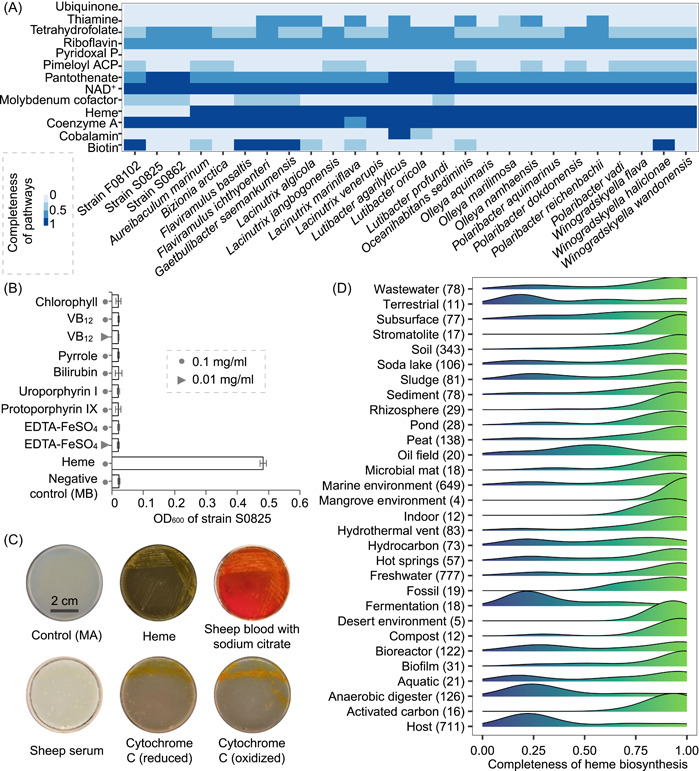
Heme as the growth factor for *Flavobacteriaceae* sp. S0825. (A) The integrity of the synthesis pathways of cofactors and vitamins in strains S0825, S0862, and F08102 and their closest relations. See Table S8 for genomic accession numbers. (B) Growth of strain S0825 in marine broth (MB) supplemented with hemin (commercial heme) and various hemin‐replacing compounds. All experiments were conducted in triplicate. Error bars represent standard errors. (C) Growth of strain S0825 on marine agar (MA) medium supplemented with sheep blood, sheep serum, and cytochrome *C*. (D) Distribution of the completeness of the heme biosynthetic pathway in different environments. The number of genomes are displayed in parentheses. The ridgeline density plots on right were used to illustrate the distribution of the completeness of heme biosynthetic pathways.

By comparing the integrity of cofactor and vitamin biosynthetic pathways in the three dependent strains and the close lineages, we found that, significantly, strains S0825, S0862, and F08102 lacked the heme synthesis pathway (Figures 4A and S6). Helper *C. aestuarii* (S08) possessed an intact heme synthesis pathway (Figure S6). In microorganisms, cytochromes are the most abundant hemoproteins. As heme in cytochromes is covalently bound to proteins, it is challenging to release unless treated with heat or proteases^27^. Therefore, we speculated that the growth‐promoting factor was heme, and the bio‐macromolecular substances that promoted the growth of strain S0825 were probably mainly composed of cytochromes.

To verify our hypothesis, we tested the growth‐promoting effect of hemin, various heme structure‐related substances (e.g., ferrous ions, synthetic precursors, synthetic intermediates, porphyrin‐containing compounds, and metabolites), and cytochrome on the growth of strain S0825. The results showed that strain S0825 was able to grow in the presence of heme but was unable to grow when EDTA‐FeSO_4_, pyrrole, uroporphyrin I, protoporphyrin IX, chlorophyll, Vitamin B_12,_ and bilirubin were substituted for hemin (Figure 4B), which was consistent with the genome analysis (Figure S6). The minimal heme concentrations required for the growth of the three *Flavobacteriaceae* strains was 0.093–0.186 µM (Figure S7 and Table S7). Strain S0825 could grow on hemoglobin‐containing sheep blood medium and cytochrome‐containing medium (Figure 4C). In addition, the anaerobic growth of strain S0825 with NO_3_^−^ as an electron acceptor also required the participation of heme (Figure S8). These results confirmed that heme and hemoproteins could function as growth factors for marine microorganisms.

### Heme auxotrophs are common in many different environments

To investigate whether heme synthesis pathway loss is also present in other uncultured bacteria from different environments, we analyzed the traits of numerous uncultured bacteria from several different habitats, using the genomes of 3760 different uncultured bacteria from the Genome Taxonomy Database (GTDB) (Table S9). Heme auxotrophs were defined as heme pathway completeness of <50%. Heme auxotrophy was common in many different phyla (e.g., *Actinomycetota*, *Bacteroidota*, *Bacillota*, *Armatimonadota*, *Chloroflexota*, *Elusimicrobiota*, *Spirochaetota*, and candidate phyla WOR‐3) and biotopes (e.g., marine, hot springs, freshwater, biofilm, anaerobic digester, fermentation, and host) (Figures 4D and S9A; Table S9). As expected, the proportion of heme auxotrophs in the host environment was significantly higher than in other nonhost habitats, which may be the reason why heme auxotrophs were always found in the host, thereby allowing us to overlook the importance of heme for uncultured microorganisms in other habitats. In addition, there were a large number of heme auxotrophs (e.g., *Patescibacteria* ^28^, DPANN^28^, *Kiritimatiellaeota* ^29^, and lactic acid bacteria^15^) in an anaerobic digester and fermentation environments, likely because bacteria in such environments rely on fermentative metabolism and lack respiration^28^. Further phylogenetic analysis revealed that heme auxotrophies were concentrated in specific subclades of different phyla. For example, in *Bacteroidota*, the great majority of heme auxotrophs were concentrated in the *Bacteroidales*, with a minority in other taxa (Figure S9B), suggesting that loss of heme biosynthesis happened independently in many lineages. These results suggest that heme auxotrophy may be a nonnegligible factor in the inability to culture many bacterial species. In conclusion, heme auxotrophy is a more common phenomenon than previously believed, and the trait is widely distributed among numerous lineages in many habitats.

**Figure 5:**
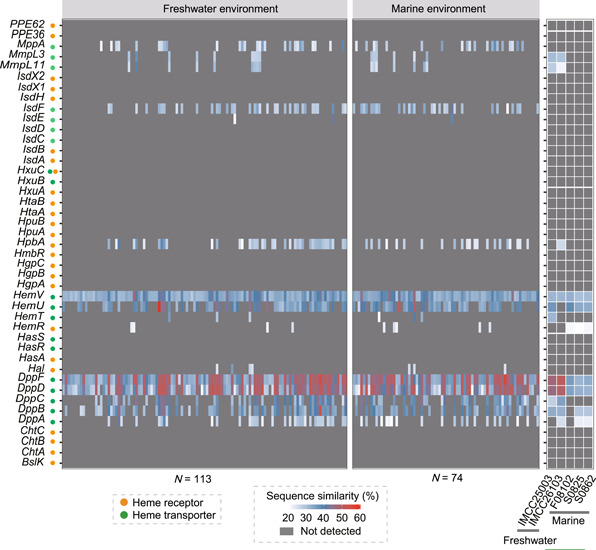
The analysis of heme uptake systems in freshwater and marine isolates. Heme uptake systems were analyzed based on 192 genomes lacking the heme synthesis pathway from freshwater and marine environments. The genes listed on the left are all reported heme uptake‐related genes in bacteria from host systems. One hundred and thirteen uncultured genomes (Table S9) and two cultured bacterial genomes (strain IMCC25003 and IMCC26103) were from freshwater environments; 74 uncultured genomes (Table S9) and three cultured bacteria genomes (strains F08102, S0825, and S0862) were from marine environments. The cut‐off of sequence similarity and coverage during local blast were 20% and 70%, respectively. The orange and green circles on the *Y*‐axis represent the heme receptor and transporter, respectively. The gray segments indicate that the related genes could not be detected. The genome Genbank accession number of strains IMCC25003 and IMCC26103 are CP029557 and CP029558, respectively.

We compared the hemin concentration requirements of cultivable heme auxotrophs from different environments. We found that bacteria in natural environments could adapt to lower heme concentrations than host‐associated bacteria, and bacteria in sediment required a higher hemin concentration than aquatic bacteria (Figure S7 and Table S7). The concentrations of total heme in sheep blood, sediment, and water were ~7 mM, 0.14–1.8 nmol/g, and 73.9–174.7 pM, respectively^30^. Bacteria in natural habitats have a low heme requirement, which may be an adaptation to oligotrophic environments. The concentrations of dissolved heme in natural waters could support the growth of bacterioplankton with heme auxotrophy; therefore, the bacterioplankton may survive in their habitats through the uptake of exogenous heme^19^. However, the heme concentration in water was well below the minimal heme concentration required by heme auxotrophs in the sediment (Figure S7 and Table S7). Our results showed that the heme‐dependent strains isolated from the sediment could grow through interactions with neighboring bacteria (Figure 3A,B). It seems likely that heme‐dependent bacteria in the sediment survive in their habitats by acquiring heme (hemoproteins) from neighboring cells. The significant differences in the heme requirements between host and nonhost microorganisms may indicate that they have different mechanisms of heme acquisition^14^, ^31^. There is almost a gap in the research on the heme acquisition pathway of heme auxotrophs in natural habitats. Therefore, using the reported heme acquisition pathways in host microorganisms as a reference, we searched the heme uptake system of heme auxotrophs in natural habitats and found their heme transport systems mainly belonged to the incomplete Dpp and Hem‐like systems (Figure 5). Heme receptors were not detected in 60% of the genomes, and three heme receptor‐like proteins (Hal, HpbA, and HemR) with 20%–40% similarity were detected in the remaining genomes (Figure 5), suggesting that bacteria in the natural environment may have unknown heme receptors.

## DISCUSSION

Bacterial species rarely live in isolation. Traditional bacterial isolation work aims to obtain a single strain, severing the interactions between species and making it difficult to get a pure culture for many microorganisms^1^, ^2^, ^3^. Preserving microbial interactions during isolation and pure‐culturing is critical for updating traditional microbial isolation methods. In this study, we developed a novel cocultivation method, the sandwich agar plate method. This technique allows pure bacterial cultures to be successfully isolated while preserving interactions with other bacteria (Figure 1).

In 1882, Robert Koch first isolated and purified *Mycobacterium tuberculosis* on a solid medium^32^. The classic plate isolation method remains irreplaceable due to its simplicity, strong visualization, and easy screening^2^. However, plate isolation relies on dilution^32^, ^33^, which significantly weakens microbial interactions. In particular, as the sample is diluted to greater degrees, rare species distributed on the plate will inevitably be lost and thus unable to perform their relevant function in the diluted community^2^, ^34^. To enable the positive interactions of rare species with other microorganisms during dilution separation, we added rare species to the sandwich layer to ensure they are not lost by dilution. Subsequent subcultivation of the isolate was also performed on sandwich agar plates (Figure 1), ensuring that individual isolates could interact with specific microorganisms even during their isolation and purification. Isolation of large numbers of uncultured and helper‐dependent bacteria confirmed that the strategy for inducing the growth of uncultured bacteria by rare species is feasible (Figure 2). In addition, many rare taxa, including the selected taxa in our study, provide a variety of “public goods” (e.g., amino acids, vitamins, cofactors, indoles, siderophores, diverse organic carbon, and organic nitrogen) (Figure S3) to facilitate the growth of other microorganisms^10^. These properties, supporting specific rare species, would play a vital helper role in the ecosystem.

The relationships between each member in the microbial communities are intricate and complex^35^, ^36^. Cross‐feeding might not be limited to isolated pairs of interacting microorganisms, as several receiver species could benefit from the metabolites of the same provider species^37^, ^38^, which is also supported by our results (Figure S2). So far, only a few cases of interspecies cooperation have been verified^36^, ^37^, ^39^. The positive multi‐species interactions verified in this study (Figure S2) could complement this aspect to some extent and provide substantial evidence for further revealing the rich network of metabolic interactions among microorganisms^35^.

Although many novel species were isolated by our method, almost all of the isolates in the experimental and control groups belonged to four common bacterial phyla (Table S3), implying that helper bacteria may not fundamentally alter the selective effect of MA medium on microbes. Despite this, adding a helper significantly facilitated the isolation of uncultured microorganisms, even for regular media. It would be better if our approach could be coupled with culturomics and diversified culture conditions^40^. In addition, it is interesting that nearly all novel *Bacillaceae* members were from cocultivation, most of which were unable to grow on MA medium (Figure 2C and Table S6). Because bacteria within the family *Bacillaceae* (phylum *Bacillota*) easily form resistant endospores, they were perceived as easy to cultivate. However, this was not the fact. In our study, many as‐yet‐undiscovered *Bacillaceae* species were isolated, and their growth depended on commensal interactions with other bacteria. This finding expands our knowledge of culturing conditions for the family *Bacillaceae*.

In this study, we investigated the promotion mechanism of an interacting pair of species and found that heme could serve as a growth factor for specific marine bacteria (Figure 4). Kim et al. found that many not‐yet‐cultured putative heme auxotrophies mainly distributed in aquatic environments^19^, whereas our findings revealed that heme auxotrophs were prevalent in many different environments (e.g., marine, hot springs, freshwater, soil, and biofilm) (Figure 4D), expanding the habitat types of heme auxotrophies. Our study further supported that heme could mediate microbial interactions in marine sediments, in turn affecting the survival and growth of bacteria, like that the zincmethylphyrins and coproporphyrins released by *Sphingopyxis* sp. enable laboratory cultivation of previously uncultured *Leucobacter* sp. through interspecies mutualism^41^. However, not all bacteria with a complete heme synthesis pathway can serve as helpers to promote the growth of heme‐dependent bacteria (Table S10), suggesting that the growth‐promoting effect of interactions could not be inferred simply by the complementarity of the genomes from both parties^42^. The number of metabolites synthesized by bacteria in the environment and whether they are released into the surrounding environment are crucial considerations. In addition, our study showed that heme‐mediated interactions require a relatively close cellular distance between the interacting parties (Figure 3A,B), supporting that some microbial interactions were distance‐dependent^43^. This also may partially explain the very high microbial community diversity of biofilm; the closeness between cells dramatically improves the opportunities for microbial interactions^44^.

We found large differences in the heme requirements of heme auxotrophy in host, sediment, and aquatic environments (minimal heme concentration: host > sediment > aquatic) (Figure S7). Heme auxotrophic strains isolated from marine sediments grew well in the medium with cytochrome *C* (Figure 4C), whereas many reported host‐associated heme auxotrophs did not^27^, ^45^. This finding suggested that bacteria in marine sediments could obtain heme from the difficult‐to‐use cytochrome *C*. The above differences may be due to differences in the heme content and origin of different habitats: (i) In the host, hemoglobin is the main source of heme^14^, and heme in this source is more readily released than the heme in cytochromes^27^, ^46^. Therefore, it was reasonable that heme auxotrophs in the host would not utilize cytochrome heme. (ii) In natural aquatic environments, the distribution of microorganisms is more dispersed^47^, ^48^, and the extremely low‐content free/dissolved heme liberated from various co‐occurring organisms maintained the growth of bacterioplankton with heme auxotrophy^19^, ^30^. The extremely low hemin requirement by bacterioplankton would result from adaptation to an extremely oligotrophic environment. (iii) Unlike the natural aquatic environments, sediment contains a dense microbial community where there is close contact between cells^47^, ^49^; in addition to a small amount of dissolved heme, this habitat contains a significant amount of intracellular hemeprotein (mainly cytochromes)^50^. Our study supported that the large number of cytochromes from neighboring bacteria could provide sufficient heme for heme auxotrophs in the sediment, resulting in a higher heme requirement for these auxotrophs in the sediment than the auxotrophs in the water column (Figure S7). Our findings provide insight into the potential niche partitioning factors of different heme auxotrophs. Furthermore, unlike the diverse heme uptake systems in host environments, the heme transport system in natural habitats consists mainly of the Dpp and Hem‐like systems (Figure 5), suggesting that both systems may be related to the adaptation of bacteria to a non‐host environment. There were no known heme acceptors found in numerous heme auxotrophs from sediments and water (Figure 5), implying that novel heme acceptors with a higher affinity for heme and the ability to bind heme in cytochromes have evolved in non‐host dependent bacteria. This heme acquisition mechanism needs to be further validated in subsequent studies.

In summary, our approach focuses on the isolation phase of the coculture process. The method will facilitate the development of effective and ecological function‐related culture/isolation strategies. These improved capabilities will allow us to learn more about bacterial biology, physiology, and ecological roles and their interactions with other members. Furthermore, we believe that employing the more ‘rare species’ as the helper in the sandwich plate could contribute to isolating more uncultured microbes, thereby digging more microbial interactions in the complex community.

## MATERIALS AND METHODS

### Preparation of sandwich agar medium and control medium

The helper organism *C. aestuarii* KCTC 12480^T^, *R. beibuensis* MCCC 1F00103^T^, *F. spongicola* KCTC 22662^T^, *S. decolorationis* JCM 21555^T^, *M. algicola* DSM 16394^T^, *S. mucosus* LMG 22090^T^, *C. marinus* JCM 19401^T^, and *A. pectinivorans* JCM 17107^T^ were collected from Korean Collection for Type Cultures (KCTC), Marine Culture Collection of China (MCCC), Japan Collection of Microorganisms (JCM), DSMZ‐German Collection of Microorganisms and Cell Cultures GmbH (DSMZ) and Belgian Coordinated Collections of Microorganisms (BCCM/LMG), respectively. All helper strains were cultivated on MA medium at 30°C. As shown in Figure 1, the production process of sandwich agar medium was as follows: (i) the MA was poured into a 9 cm plate; (ii) the helper was spread on the MA covering a 6–7 cm diameter circular area. These helper organisms were cultured at 30°C until the colonies covered the spread area. (iii) 1/10 MA with 4% agar (2% NaCl) was poured on the helper bacteria layer. The control medium was a double‐layer agar medium without a helper strain.

### Marine sediment sampling, incubation, and the classification of isolated strains

Offshore sediments were collected from the Jingzi Port of Weihai, Shandong Province, China (122°7′38.80″E, 37°33′57.60″N) on 1 October 2019. The samples were processed by series dilution, then spread‐plated onto various sandwich agar media and the control medium, and incubated aerobically at 25°C for 3 weeks. Subcultivation of isolates was also performed on sandwich agar plates. The amplification and classification of the 16 S rRNA genes were performed following the procedure of Liu et al.^51^ and using the EZBioCloud server^52^, respectively.

### High‐throughput sequencing

All colonies on each plate were resuspended in phosphate‐buffered saline (PBS). The DNA was extracted using the Fast DNA SPIN Kit for Soil according to the manufacturer's instructions (MP Biomedicals). The V3–V4 region of 16 S rRNA genes were sequenced on the Illumina MiSeq platform using the primer pair 338 F and 806 R (Majorbio Bio‐Pharm Technology Co., Ltd.). The operational taxonomic units (OTUs) were clustered based on 97% sequence identity and annotated against the SILVA 138 database^53^.

### DNA extraction, sequencing, and genomic analysis

The genomic DNA of the isolates was extracted with a bacterial genomic DNA mini kit (Macherey‐Nagel NucleoSpin®). The genomic DNA was sequenced by Genewiz using an Illumina Hiseq PE150 platform. Assembly was performed using Velvet^54^. We confirmed 3760 genomes of uncultured bacteria according to the Genome Taxonomy Database (GTDB)^55^ (Table S9) and then downloaded these confirmed genomes from the NCBI database for downstream analysis. The protein‐coding gene regions were identified using Prodigal^56^ with default settings. The function annotation of protein‐coding genes was performed using KofamKOALA^57^. The GTDB‐tk v0.3.2^58^ was used for phylogenomic analysis. We also employed FastTree2^59^ for phylogenetic analysis, and the tree visualization was created on iTOL^60^.

### Growth‐promotion assay

Every isolate was diluted and spread‐plated on MA, and then the helper was resuspended in 3% NaCl solution at a high density and spotted (2 μl) on the plate spread with the isolate. The co‐cultures were incubated for 2–5 days at 28°C and observed daily. Only the isolates that grew, or displayed improved growth, around the spotted helpers were marked as a positive strain.

The helped strains *Bacillaceae* sp. F26177 and S0848, *Rhodobacteraceae* sp. F26174, and *Flavobacteriaceae* sp. S0825 were plated on the top in the transwell (Corning) with 0.4 μm inserts, respectively; the helper strains *C. aestuarii* S08 and *S. decolorationis* S26 were plated on the bottom in the transwell, respectively. The cocultures were incubated at 28°C for 50 h, and the cell biomass of the helped strains was determined by measuring the optical density (600 nm).

### The testing to confirm heme as the most critical growth factor

The growth curve of *C. aestuarii* was determined (Figure S10). Cell suspensions were collected at 4.5 h (Mid‐log phase), 9.5 h (End‐log phase), and 15 h (Stationary phase), respectively. The supernatants and cell fractions were separated by centrifugation (10,000*g*, 10 min). The supernatant was filter‐sterilized (0.22 μm; Millipore) and used to cultivate strain S0825. 200 ml of cell‐free supernatant was concentrated using the centrifugal filter devices 10 kDa (5000*g*, 30 min). The concentrated fraction was adjusted with fresh medium to 2 ml and used to cultivate strain S0825.

The cell fractions of *C. aestuarii* were re‐suspended in MB (measuring cell biomass by OD_600_) and disrupted by ultrasonication (Sonics VC751). The resulting lysates were centrifuged at 12,000*g* for 30 min at 4°C. The cell lysate solution was filter‐sterilized (0.22 μm; Millipore) and used to cultivate strain S0825. The cell‐free lysate of *C. aestuarii* was filtered using 10 kDa centrifugal filter unit (Millipore) (5000*g*, 30 min). The retentate (>10 kDa) and flow‐through (<10 kDa) were both collected. Each fraction was adjusted with fresh medium to the original volume and used to cultivate strain S0825.

Bacterial crude polysaccharides were extracted according to the method of Ai et al^61^. One hundred milliliters of the cell lysate of *C. aestuarii* was heated in boiling water and centrifuged at 10,000*g*, 4°C for 10 min. 80% (w/v) trichloroacetic acid was added to the precooled supernatant to a final concentration of 7% (w/v) and kept at 4°C for 12 h. After centrifugation (10,000*g*, 4°C for 10 min), three volumes of cold ethanol were added to the supernatant and then incubated overnight at 4°C. The precipitate collected by centrifugation was redissolved in Milli‐Q water and dialyzed (cut‐off 14 kDa) against Milli‐Q water for 3 days. The retentate was lyophilized, and then white crude polysaccharides were obtained. The crude polysaccharides were resuspended in MB (final concentration of crude polysaccharides: 1 mg/ml) and filter‐sterilized (0.22 μm; Millipore). Nucleic acids of the cell lysate of *C. aestuarii* were extracted with a bacterial genomic DNA mini kit. During the extraction process, RNase was not added, and the nucleic acid was dissolved in Milli‐Q water and lyophilized. The nucleic acids were resuspended in MB (final concentration of nucleic acids: 1 mg/ml) and filter‐sterilized (0.22 μm, Millipore). For the denaturation of protein in the cell lysate solution of *C. aestuarii*, proteinase K was added to the cell lysate solution to a final concentration of 20 µg/ml and kept at 58°C for 15 min. A separate portion of the cell lysate solution was heated in boiling water for 10 min. The MB with polysaccharide or nucleic acids extracted from the cell lysate solution of *C. aestuarii* and cell lysate solution of *C. aestuarii* treated with proteinase K or heating were used to cultivate strain S0825.

Heme was dissolved in 0.1 M NaOH. Bilirubin and uroporphyrin were dissolved in 0.02 M NaOH. Protoporphyrin IX was dissolved in a 1:1 (v/v) mixture of ethyl alcohol and 0.2 M NaOH. Vitamin B_12_ and cytochrome *C* (reduced/oxidized) were dissolved in distilled Milli‐Q water. Chlorophyll was dissolved in absolute ethyl alcohol. The solution of EDTA‐FeSO_4_ was a 1:1 (v/v) mixture of 35.971 mM EDTA‐2Na and 35.971 mM FeSO_4_•7H₂O. The solutions of hemin, protoporphyrin IX, uroporphyrin, bilirubin, and Chlorophyll were considered sterile as prepared. The solutions of pyrrole, vitamin B_12_, cytochrome *C*, and EDTA‐FeSO_4_ were filter sterilized. The commercial sheep blood with sodium citrate and sheep serum was sterile. These stock solutions of hemin and hemin‐replacing substances were added in sterile marine agar/broth 2216E to culture strain S0825. The concentration of compounds: sheep blood and sheep serum: 5% (v/v); hemin, bilirubin, uroporphyrin, protoporphyrin IX, pyrrole, chlorophyll, and cytochrome C: 0.1 mg/ml; vitamin B_12_ and EDTA‐FeSO_4_: 0.1 mg/ml and 0.01 mg/ml, respectively.

### Nontargeted metabolomics analysis of extracellular secretions (LMWOS)

The supernatant (logarithmic growth phase) was freeze‐dried and resuspended in an extraction solution (methanol: water = 4:1 [v:v]). The supernatant metabolites were extracted by ultrasonication for 30 min at low temperature (5°C, 40 KHz). After centrifugation (13,000*g*, 15 min, 4°C), the supernatant was used for LC‐MS detection (AB SCIEX). The full scan and fragment spectra were processed using ProgenesisQI (Waters Corporation). Metabolite comparisons were performed using the Human Metabolome Database (HMDB) (http://www.hmdb.ca/) and Metabolite and Tandem MS Database (METLIN) (https://metlin.scripps.edu/).

## AUTHOR CONTRIBUTIONS

**Jing Zhang**: Data curation (equal); formal analysis (equal); writing—original draft (lead); writing—review and editing (equal). **Qi‐Yun Liang**: Data curation (equal); formal analysis (equal); software (equal); visualization (lead); writing—review and editing (equal). **Da‐Shuai Mu**: Investigation (equal); supervision (equal). **Fengbai Lian**: Formal analysis (supporting). **Ya Gong**: Formal analysis (supporting). **Mengqi Ye**: Formal analysis (supporting). **Guan‐Jun Chen**: Supervision (supporting). **Yuqi Ye**: Formal analysis (supporting). **Zong‐Jun Du**: Conceptualization (lead); methodology (lead); project administration (lead); supervision (lead).

## ETHICS STATEMENT

The study in this article did not involve any trials on humans or animals.

## CONFLICT OF INTERESTS

The authors declare no conflict of interests.

## Supporting information

Supporting information.

Table S1 Taxa_otu percents in offshore sediment samples from Jingzi Harbor based on 16 S high‐throughput sequencing.

Table S2 Abundance of helper species in sediment samples from Jingzi Harbor.

Table S3 The detailed information of each isolate (ending with note).

Table S4 ANI value between the isolates and their closest species.

Table S5 OTU taxonomy.

Table S6 The results of growth‐promotion assay.

Table S7 The minimal heme concentrations required for the growth of bacteria in different habitats.

Table S8 Genomic accession numbers of strains S0825, S0862, F08102 and related type species.

Table S9 The genomes list of heme biosynthesis pathway analysis.

Table S10 Growth‐promoting effect of helpers with complete heme synthesis pathway on Flavobacteriaceae sp. S0825.

Supporting information.

Supporting information.

Supporting information.

Supporting information.

Supporting information.

Supporting information.

Supporting information.

Supporting information.

Supporting information.

Supporting information.

## Data Availability

The 16S rRNA gene data sets generated during this study have been deposited in the Sequence Read Archive under accession no. SRP427113 for 30 samples. The list of 30 runs under SRP427113, R scripts, and raw data are available on GitHub at https://github.com/2015qyliang/SandwichAgarPlate.
