# Supplementary material for: Cultivating the uncultured: Harnessing the “sandwich agar plate” approach to isolate heme‐dependent bacteria from marine sediment
Source: mLife. 2024 Jan 18;3(1):143–55. doi: 10.1002/mlf2.12093 (PMC11139205; doi:10.1002/mlf2.12093)
Supplement: Supplementary file 1 — Supporting information. [file MLF2-3-143-s002.docx]

**Supplementary Materials**

**Co-culture based on ‘sandwich agar plate’ insight into heme as a growth factor of uncultured bacteria in marine sediment**

Jing Zhang ^a,b†^, Qi-Yun Liang ^b†^, Da-Shuai Mu ^a,b,c†^, Feng-Bai Lian ^b^, Ya Gong ^a,b^, Meng-Qi Ye ^a,b^, Guan-Jun Chen ^a,b^, Yu-Qi Ye ^b^, Zong-Jun Du ^a,b,c *^

^a^ State Key Laboratory of Microbial Technology, Institute of Microbial Technology, Shandong University, Qingdao, 266237, China

^b^ Marine College, Shandong University, Weihai, 264209, China

^c^ Shandong University-Weihai Research Institute of Industrial Technology, Weihai, 264209, China

† These authors contributed equally to this work.

* Address correspondence to:

Zong-Jun Du, E-mail: [duzongjun@sdu.edu.cn](mailto:duzongjun@sdu.edu.cn); Tel.: +86-0631-5688303. Mailing address: Marine College, Shandong University, No. 180, Wenhua Xilu, Weihai, 264209, China

This file includes:

**Figure legends S1 to S10**

**Figure S1 The cultivable bacterial community compositions on 8 sandwich agar plates and the control plate (using high-throughput sequencing).**

(A) Partial least squares discriminant analysis (PLS-DA) of bacterial communities at the OTU level. (B) The cultivable bacterial community compositions at the phyla level.

Note: Each group had three biological replicates. The data in figure b show the mean of 3 replicates. CG (Control group): control medium; S08: *C. aestuarii* S08 medium; S20: *F. spongicola* S20 medium; S11: *R. beibuensis* S11 medium; S26: *S. decolorationis* S26 medium; S47: *M. algicola* S47 medium; S60: *S. mucosus* S60 medium; S63: *C. marinus* S63 medium; S64: *A. pectinivorans* S64 medium.

**Figure S2 Complex interactions between positive species and helpers and the co-culture testing in the liquid media.**

(A), A maximum-likelihood phylogenetic tree of 115 positive species based on the growth promotion assay is displayed at the center. The taxonomic family of positive species is colored randomly as the background of tree-leaf labels and clades. 1st annuluses represent the corresponding helper for each positive strain, and eight helpers are colored randomly. 2nd to 9th annuluses represent the growth-promoting effect of different helpers on each positive bacteria. The colorful segments represent that the helper could promote the growth of the positive bacteria; colorless segments represent the helper couldn’t promote the growth of the positive bacteria. (B), The co-culture illustration of helper and helped strains in the liquid media by using the Transwell (0.4 μm pore polyester membrane). The orange and the grey ellipses represented the helped and helper strains, respectively. The blue square represented the marine broth 2216 (MB). (C) The growth changes of co-culture in the liquid media by using the transwell. Strains F26174, F26177, S0825 and S0848 were the helped strains, and strains S08 and S26 were the helper strains.

Note: the helper strain, S08: *C. aestuarii*; S11: *R. beibuensis*; S20: *F. spongicola*; S26: *S. decolorationis*; S47: *M. algicola*; S60: *S. mucosus*; S63: *C. marinus*; S64: *A. pectinivorans*.

**Figure S3 Metabolome analysis.**

Heatmaps indicating significantly up-regulated metabolites in spent-culture supernatants of different helper cells vs. control medium. The color scale represents each up-regulated metabolite's fold change (helper/CK). The white color scale represents that the metabolite was not detected. Metabolites with fold-change values >1 are considered to be up-regulated metabolites. All samples were from six biological replicates.

Note: S08: *C. aestuarii*; S20: *F. spongicola*; S26: *S. decolorationis.*

**Figure S4 The growth-promotion assay of some *Bacillaceae* species.**

The *Bacillaceae* species was spread over the entire MA, and 2 μL culture of the helper was then spotted on the same plate.

Note: helper S08: *C. aestuarii*; helper S20: *F. spongicola*; helper S26: *S. decolorationis*; helper S60: *S. mucosus*; helper S64: *A. pectinivorans*.

**Figure S5 Growth of *Flavobacteriaceae* sp. S0825 in concentrated** **spent-culture supernatant of *C. aestuarii.***

>10KDa fraction in the spent-culture supernatant of *C. aestuarii* was concentrated 100-fold using centrifugal filter devices of 10KDa and resuspended in MB. The cell growth of strain S0825 was determined by measuring the optical density (600 nm) of the cells grown. All the experiments were conducted with three repeats. Error bars represent standard errors. ***p < 0.001.

**Figure S6** **The analysis of heme biosynthesis pathways.**

The heme synthesis pathways of *Flavobacteriaceae* sp. S0825, S0862, and F08102 and helper *C. aestuarii*. The white box represents that the gene is not found in the genome of the strain.

**Figure S7 The minimal hemin growth concentration.**

The minimal heme concentrations required for the growth of the cultured bacteria with heme auxotrophy in the host (e.g., human and chick), freshwater, and sediment (Table S8).

**Figure S8 Growth of *Flavobacteriaceae* sp. S0825 under anaerobic conditions.**

Growth of *Flavobacteriaceae* sp. S0825 on MA with or without NO^3-^ or hemin under anaerobic conditions (10% H_2_, 10% CO_2_, and 80% N_2_) were determined after incubation for 14 days. A facultative anaerobic bacterium (*Pricia* sp. D202) was a positive control, and a strictly aerobic bacteria (*Fulvivirga* sp. 1062) was a negative control.

**Figure S9 Distribution of the completeness of the heme biosynthetic pathway in different taxonomic groups.**

(A) Distribution of the completeness of the heme biosynthetic pathway in 3,760 uncultured genomes with different phylum (genomes ≥ 10; Table S9). (B) Distribution of the completeness of the heme biosynthetic pathway in uncultured taxonomic class groups. The maximum-likelihood phylogenetic trees of the representative genomes in uncultured taxonomic class groups are shown on left. The clades of the representative groups in phylogenetic trees are randomly colored. The ridgeline density plots were used to illustrate the distribution of the completeness of heme biosynthetic pathways.

**Figure S10 Time courses of cell growth (OD_600_) in *C. aestuarii* S08.**

The growth curve of *C. aestuarii* S08 was determined by measuring the optical density (600 nm) of the cells grown in MB from 0 h to 30 h at 28 °C. All the experiments were conducted with three repeats. Error bars represent standard errors.
