# Supplementary figures and images for: Cultivating the uncultured: Harnessing the “sandwich agar plate” approach to isolate heme‐dependent bacteria from marine sediment
Source: mLife. 2024 Jan 18;3(1):143–55. doi: 10.1002/mlf2.12093 (PMC11139205; doi:10.1002/mlf2.12093)

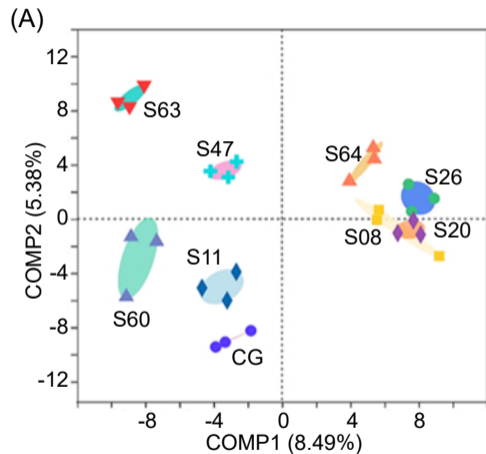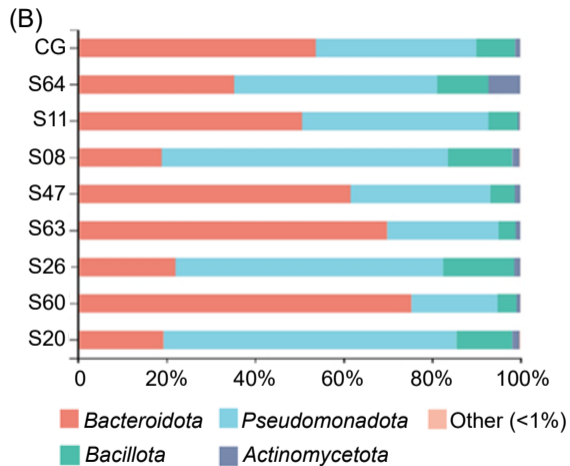

Supplement: Supplementary file 12 — Supporting information. [file MLF2-3-143-s019.pdf]

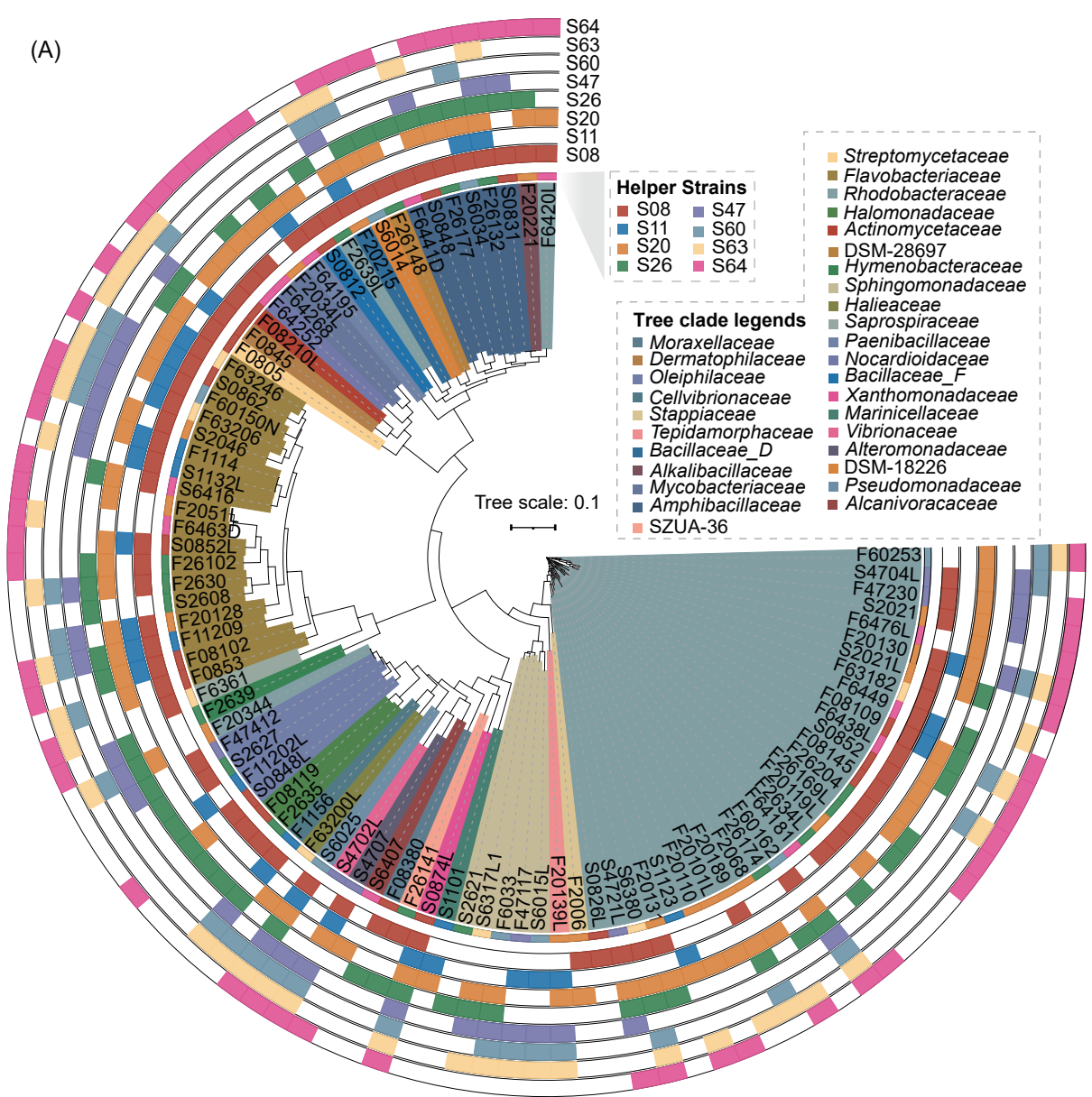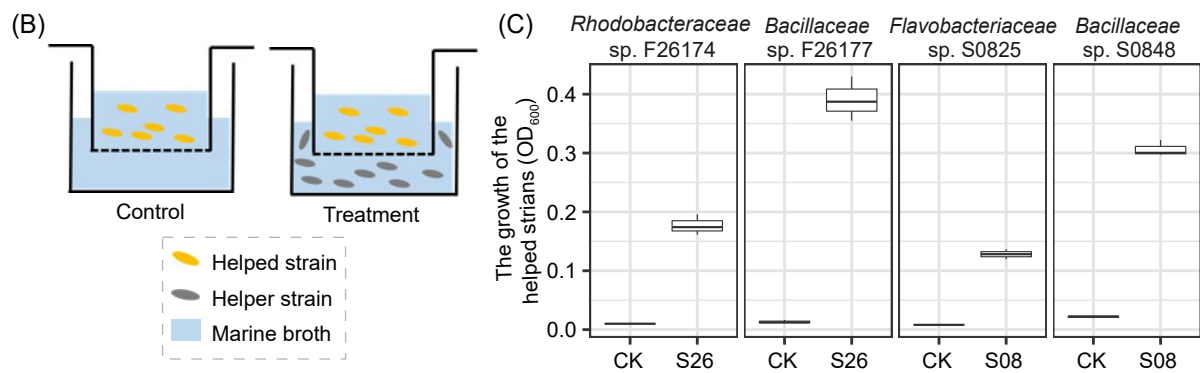

Supplement: Supplementary file 13 — Supporting information. [file MLF2-3-143-s016.pdf]

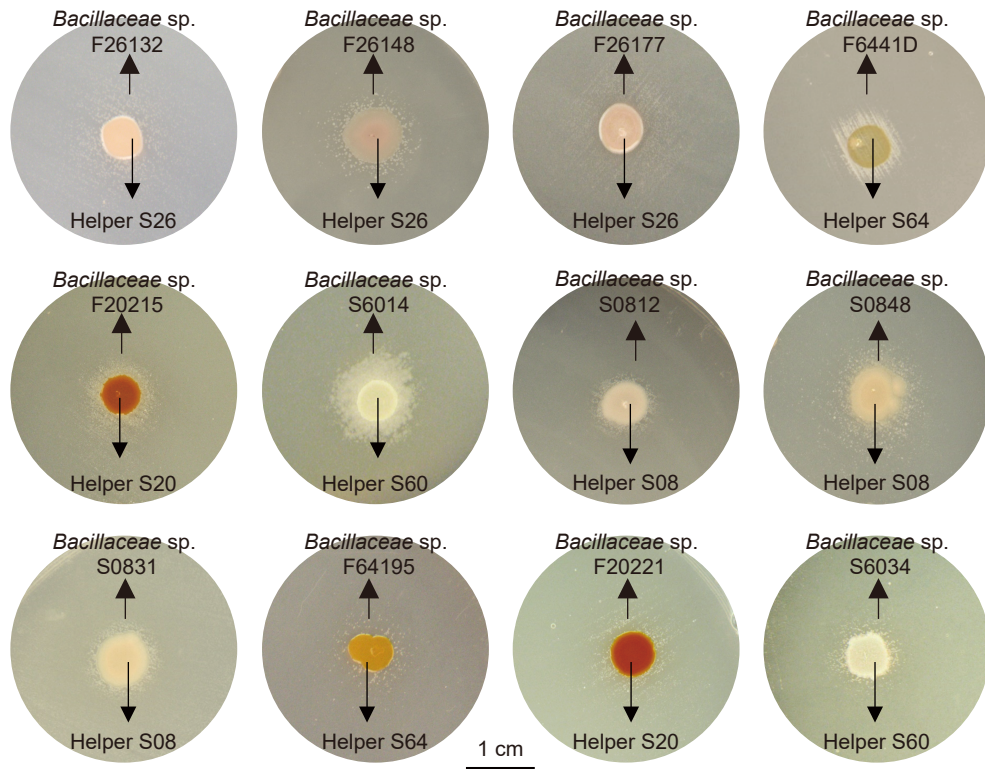

Supplement: Supplementary file 15 — Supporting information. [file MLF2-3-143-s011.pdf]

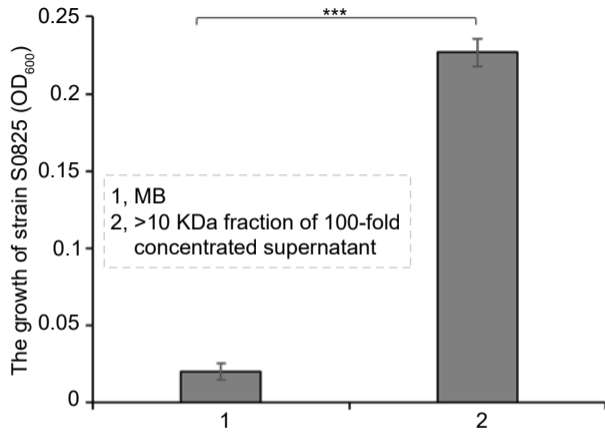

Supplement: Supplementary file 16 — Supporting information. [file MLF2-3-143-s007.pdf]

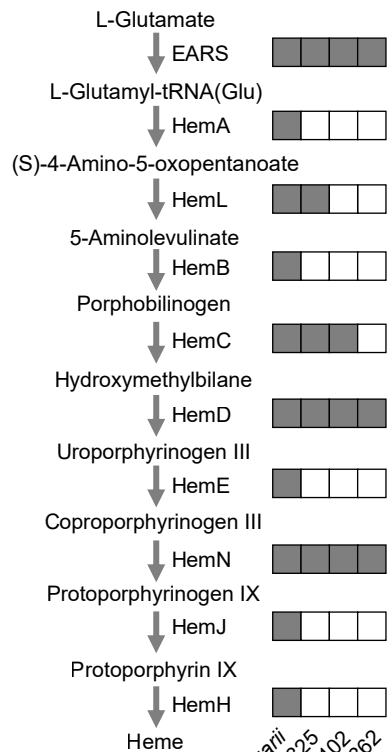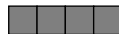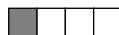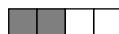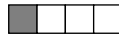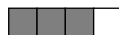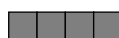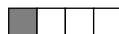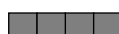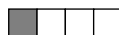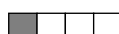

*C. aestuarii*  
Strain S0825  
Strain F08102  
Strain S0862

Supplement: Supplementary file 17 — Supporting information. [file MLF2-3-143-s013.pdf]

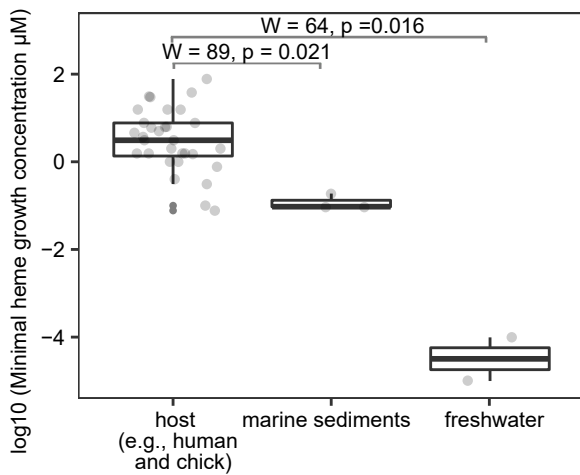

Supplement: Supplementary file 18 — Supporting information. [file MLF2-3-143-s018.pdf]

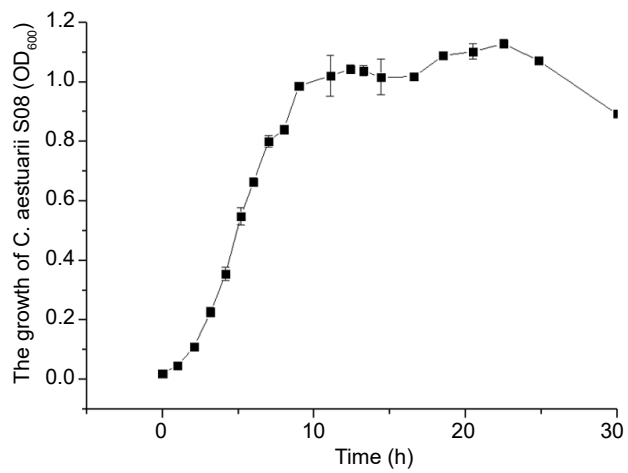

Supplement: Supplementary file 21 — Supporting information. [file MLF2-3-143-s003.pdf]
