# Supplementary material for: Cultivating the uncultured: Harnessing the “sandwich agar plate” approach to isolate heme‐dependent bacteria from marine sediment
Source: mLife. 2024 Jan 18;3(1):143–55. doi: 10.1002/mlf2.12093 (PMC11139205; doi:10.1002/mlf2.12093)
Supplement: Supplementary file 14 — Supporting information. [file MLF2-3-143-s010.pdf]

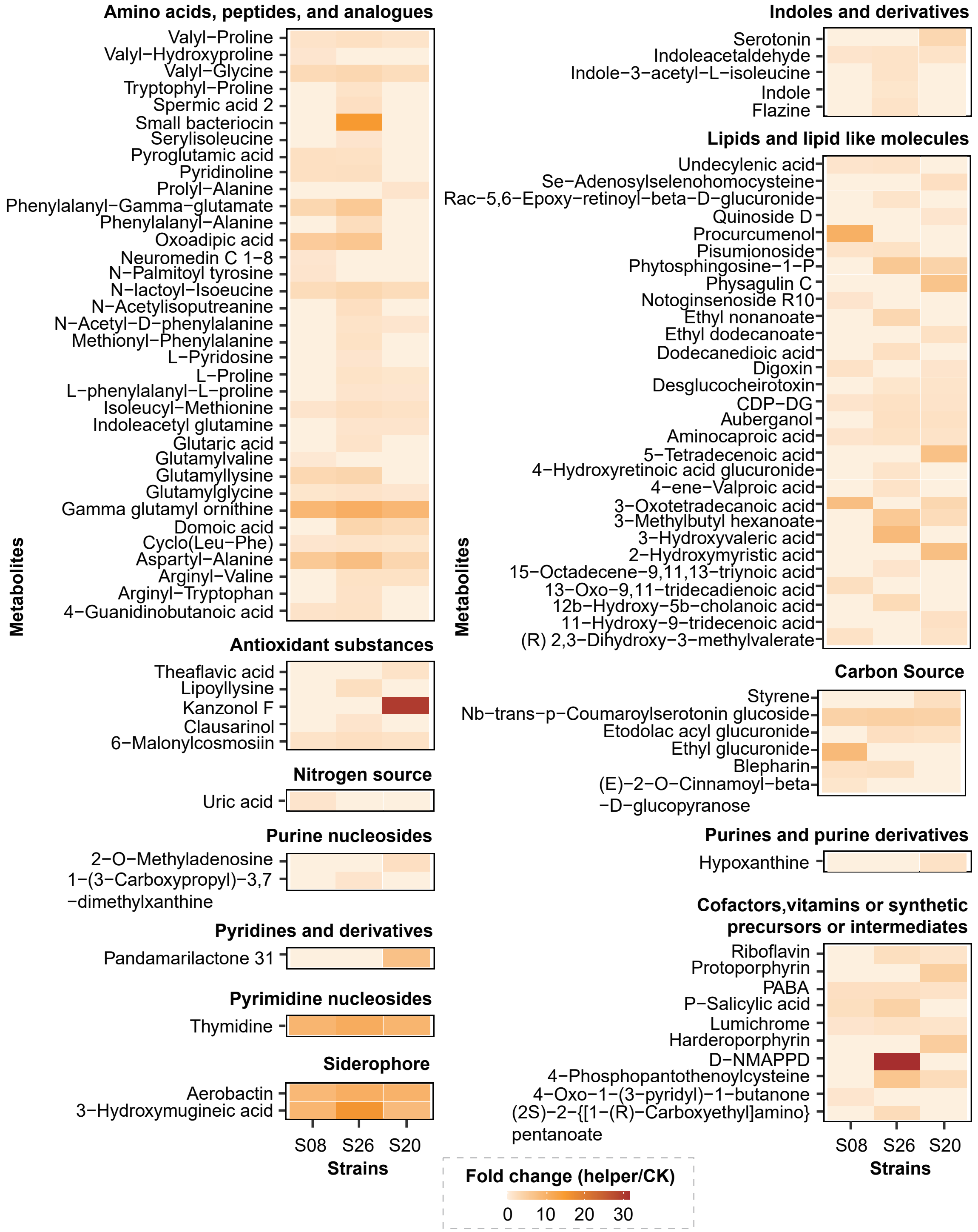
Amino acids, peptides, and analogues
Valyl−Proline
Valyl−Hydroxyproline
Valyl−Glycine
Tryptophyl−Proline
Spermic acid 2
Small bacteriocin
Serylisoleucine
Pyroglutamic acid
Pyridinoline
Prolyl−Alanine
Phenylalanyl−Gamma−glutamate
Phenylalanyl−Alanine
Oxoadipic acid
Neuromedin C 1−8
N−Palmitoyl tyrosine
N−lactoyl−Isoeucine
N−Acetylisoputreanine
N−Acetyl−D−phenylalanine
Methionyl−Phenylalanine
L−Pyridosine
L−Proline
L−phenylalanyl−L−proline
Isoleucyl−Methionine
Indoleacetyl glutamine
Glutaric acid
Glutamylvaline
Glutamyllysine
Glutamylglycine
Gamma glutamyl ornithine
Domoic acid
Cyclo(Leu−Phe)
Aspartyl−Alanine
Arginyl−Valine
Arginyl−Tryptophan
4−Guanidinobutanoic acid
Metabolites
Antioxidant substances
Theaflavic acid
Lipoyllysine
Kanzonol F
Clausarinol
6−Malonylcosmosiin
Nitrogen source
Uric acid
Purine nucleosides
2−O−Methyladenosine
1−(3−Carboxypropyl)−3,7
−dimethylxanthine
Pyridines and derivatives
Pandamarilactone 31
Pyrimidine nucleosides
Thymidine
Siderophore
Aerobactin
3−Hydroxymugineic acid
S08
S26
S20
Strains
Indoles and derivatives
Serotonin
Indoleacetaldehyde
Indole−3−acetyl−L−isoleucine
Indole
Flazine
Lipids and lipid like molecules
Undecylenic acid
Se−Adenosylselenohomocysteine
Rac−5,6−Epoxy−retinoyl−beta−D−glucuronide
Quinoside D
Procurcumenol
Pisumionoside
Phytosphingosine−1−P
Physagulin C
Notoginsenoside R10
Ethyl nonanoate
Ethyl dodecanoate
Dodecanedioic acid
Digoxin
Desglucocheirotoxin
CDP−DG
Auberganol
Aminocaproic acid
5−Tetradecenoic acid
4−Hydroxyretinoic acid glucuronide
4−ene−Valproic acid
3−Oxotetradecanoic acid
3−Methylbutyl hexanoate
3−Hydroxyvaleric acid
2−Hydroxymyristic acid
15−Octadecene−9,11,13−triynoic acid
13−Oxo−9,11−tridecadienoic acid
12b−Hydroxy−5b−cholanoic acid
11−Hydroxy−9−tridecenoic acid
(R) 2,3−Dihydroxy−3−methylvalerate
Metabolites
Carbon Source
Styrene
Nb−trans−p−Coumaroylserotonin glucoside
Etodolac acyl glucuronide
Ethyl glucuronide
Blepharin
(E)−2−O−Cinnamoyl−beta
−D−glucopyranose
Purines and purine derivatives
Hypoxanthine
Cofactors,vitamins or synthetic
precursors or intermediates
Riboflavin
Protoporphyrin
PABA
P−Salicylic acid
Lumichrome
Harderoporphyrin
D−NMAPPD
4−Phosphopantothenoylcysteine
4−Oxo−1−(3−pyridyl)−1−butanone
(2S)−2−{[1−(R)−Carboxyethyl]amino}
pentanoate
S08
S26
S20
Strains
Fold change (helper/CK)
0
10
20
30
