# Supplementary material for: Cultivating the uncultured: Harnessing the “sandwich agar plate” approach to isolate heme‐dependent bacteria from marine sediment
Source: mLife. 2024 Jan 18;3(1):143–55. doi: 10.1002/mlf2.12093 (PMC11139205; doi:10.1002/mlf2.12093)
Supplement: Supplementary file 19 — Supporting information. [file MLF2-3-143-s021.pdf]

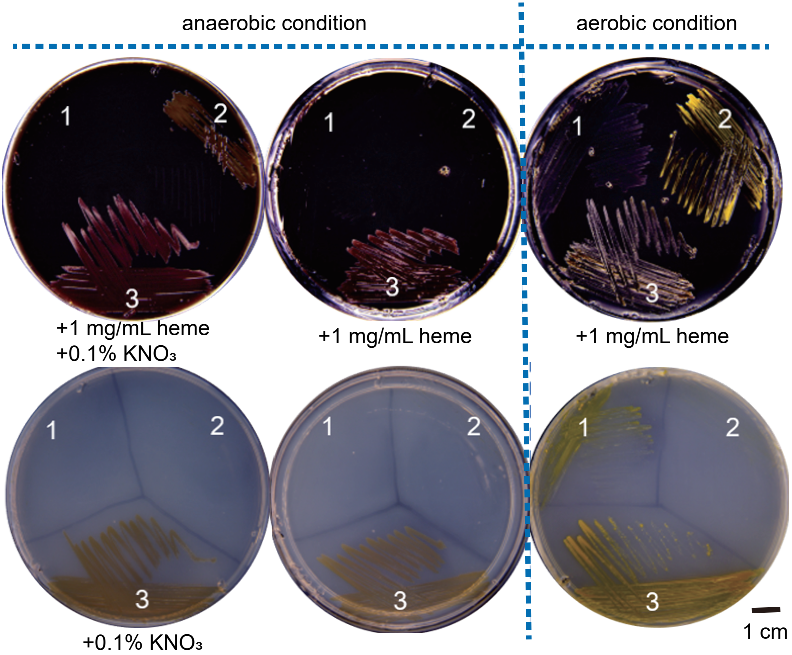

1: *Fulvivirga* sp. 1062 (Negative control, strict aerobic bacteria)
2: *Flavobacteriaceae* sp. S0825
3: *Pricia* sp. D202 (Positive control, facultative aerobic bacteria)
